# Long COVID outcomes following omicron wave in non-hospital population

**DOI:** 10.3389/fpubh.2024.1377866

**Published:** 2024-03-15

**Authors:** Wang Ruiyin, Jia Qi, Wang Tingting, Yan Yuqin, Jia Yan, Peng Kun

**Affiliations:** ^1^Department of Respiratory, Beijing Longfu Hospital, Beijing, China; ^2^Department of Office of the Hospital, Beijing Longfu Hospital, Beijing, China

**Keywords:** long COVID, PASC, omicron, non-hospital population, risk factor

## Abstract

**Background:**

The persistence of symptoms or the development of new symptoms following a diagnosis of SARS-CoV-2 has given rise to a multifaceted clinical condition referred to as “long COVID” (LC). The understanding of LC among China’s non-hospitalized population continues to be insufficient. This investigation was designed to evaluate the protracted consequences amongst this demographic, as well as to identify the associated risk factors.

**Methods:**

This research constitutes a prospective cohort study focusing on non-hospitalized individuals, aged between 18 and 59, who have been positively diagnosed with COVID-19. Each participant was subjected to a sequence of questionnaire-based surveys, designed to evaluate symptoms as well as the status of depression and anxiety. A logistic regression model, adjusted for multiple variables, was employed to scrutinize the correlation between demographic elements, lifestyle attributes, and health-related risk factors in relation to conditions and symptoms post COVID-19 infection.

**Results:**

A total of 706 individuals participated in the 3 months follow-up, with 620 continuing on to the 6 months follow-up. The median age was 35 (28, 43) years, and 597 (85%) are female. Upon follow-up, Compared with patients without LC, patients with LC have a higher proportion of females (420 (87%) vs. 177 (79%); *p* = 0.010), were older (35 (29, 44) years vs. 33 (27, 41) years; *p* = 0.010) and have more comorbidities. Out of all participants, 483 (68.4%) reported experiencing at least one symptom at the 3 months mark, while 49.7% reported symptoms persisting at the 6 months mark. At the 3 months follow-up, the most prevalent persistent symptoms were cough (46%), fatigue (38%), and shortness of breath (34%). By the 6 months follow-up, fatigue (25%), shortness of breath (22%), and sleep disorders (16%) were the most commonly reported symptoms. Anxiety and depression were consistently reported as prevalent symptoms throughout the follow-up period. Most patient symptoms fade over time, with the quickest decreases observed in cough (from 46 to 9%), expectoration (from 26 to 6.3%), smell disorder (from 16 to 3.9%), and taste disorder (from 18 to 3.5%). Male and those possessing advanced educational qualifications exhibit a decreased susceptibility to the sustained incidence of coughing. Conversely, older age and the presence of comorbidities were identified as risk factors for persistent fatigue and shortness of breath.

**Conclusion:**

In the after of COVID-19, it has been observed that the majority of patient symptoms tend to decrease over time. The primary residual symptoms noticed after a 6 month follow-up were fatigue, dyspnea, and sleep disturbances. However, it’s noteworthy that the risk factors associated with these symptoms exhibit subtle variations. Furthermore, psychological sequelae, namely depression and anxiety, are frequently reported among COVID-19 survivors.

## Introduction

Long COVID (LC), or post-acute sequelae of SARS-CoV-2 infection (PASC), has become a substantial public health issue. LC is a term frequently employed to delineate symptoms that persist or manifest subsequent to an acute diagnosis of SARS-CoV-2, extending beyond the initial four-week period (1). This condition, frequently experienced by patients who have recovered from acute SARS-CoV-2 infection, is characterized by lingering symptoms such as fatigue, muscular weakness, dyspnea, arthralgia, and neurological complications (2, 3). The prevalence of LC remains uncertain; however, conservative estimates suggest that it affects approximately 10% of non-hospitalized survivors, with a higher proportion among hospitalized individuals (4, 5), this condition significantly deteriorates the quality of life and imposes a considerable economic burden (6).

Regarding the long-term consequences of the Omicron variant, preliminary evidence suggests a trend towards less severity and shorter duration compared to the Delta variant and other strains. Arjun’s et al. research in India indicates an 8.2% risk of LC following an Omicron infection, markedly lower than the risk associated with the Delta variant (7). Consistently, Antonelli et al. reported a lower incidence of LC in Omicron cases (4.8%) than in Delta cases (10.8%) (8). In China, the first wave of the Omicron variant occurred in December 2022, yet the long-term effects on non-hospitalized individuals remain largely undetermined.

Therefore, it is imperative to evaluate the enduring repercussions of COVID-19 in non-hospitalized patients and to identify potential risk factors. This will equip healthcare providers with the necessary information to effectively manage LC and its impact on patients and their families. Additionally, it will contribute to the growing body of knowledge regarding LC in non-hospitalized populations.

## Methods

### Study design and participants

In this prospective cohort study, non-hospitalized patients confirmed with COVID-19 infection through reverse transcriptase polymerase chain reaction (RT-PCR) or COVID-19 antigen testing were recruited from Longfu Hospital in Beijing, China. The study population comprises hospital staff, encompassing both permanent and contingent employees, together with police department personnel who collaborate with the medical institution. The inclusion criteria encompassed individuals aged between 18 and 59 years. Subjects were excluded if they declined participation, failed follow-up, were unable to articulate their symptoms, or experienced COVID-19 reinfection during the follow-up period. The data collection spanned from December 2022 to August 2023. The study received approval from the Research Ethics Commission of Beijing Longfu Hospital (LFYYLL-2023-01), and written informed consent was obtained from all participants.

### Data management and outcome measurement

The acute phase data incorporated demographics (including age, gender, body mass index (BMI), educational background, and smoking and drinking habits), symptoms, comorbidities, and chest computed tomography. Subsequent clinical follow-ups of all study participants were conducted via telephone consultations and in-person appointments at 3- and 6 months intervals after diagnosis. During each encounter, the Long-term Follow-up Case Report Form (CRF), based on the World Health Organization’s CRF for post-COVID conditions, was utilized to gather information regarding the patient’s current health status and any lingering symptoms. The Self-Rating Depression Scale (SDS) and Self-Rating Anxiety Scale (SAS) were employed to assess the prevalence and intensity of depressive and anxious symptoms. All follow-up information was ultimately compiled into Microsoft Excel for streamlined storage and efficient management.

### Statistical analysis

We undertook descriptive statistical analysis to assess the baseline characteristics and enduring health implications of COVID-19. Both continuous and categorical variables were represented as median (interquartile range (IQR)) and frequency (percentage), respectively. For group comparisons, we deployed the Chi-squared test or Fisher’s exact test in conjunction with the Wilcoxon rank-sum test. A *p*-value below 0.05 was deemed statistically significant.

To explore and ascertain the odds ratios (ORs) and 95% confidence intervals (CIs) for associations between demographic attributes, comorbidities, and persistent symptoms, univariable and multivariable logistic regression analyses were conducted. Our final analysis incorporated all participants for whom the variables of interest were accessible, excluding the imputation of missing data. All statistical evaluations were executed using R software, version 4.0.2.^1^

## Results

**Figure 1:**
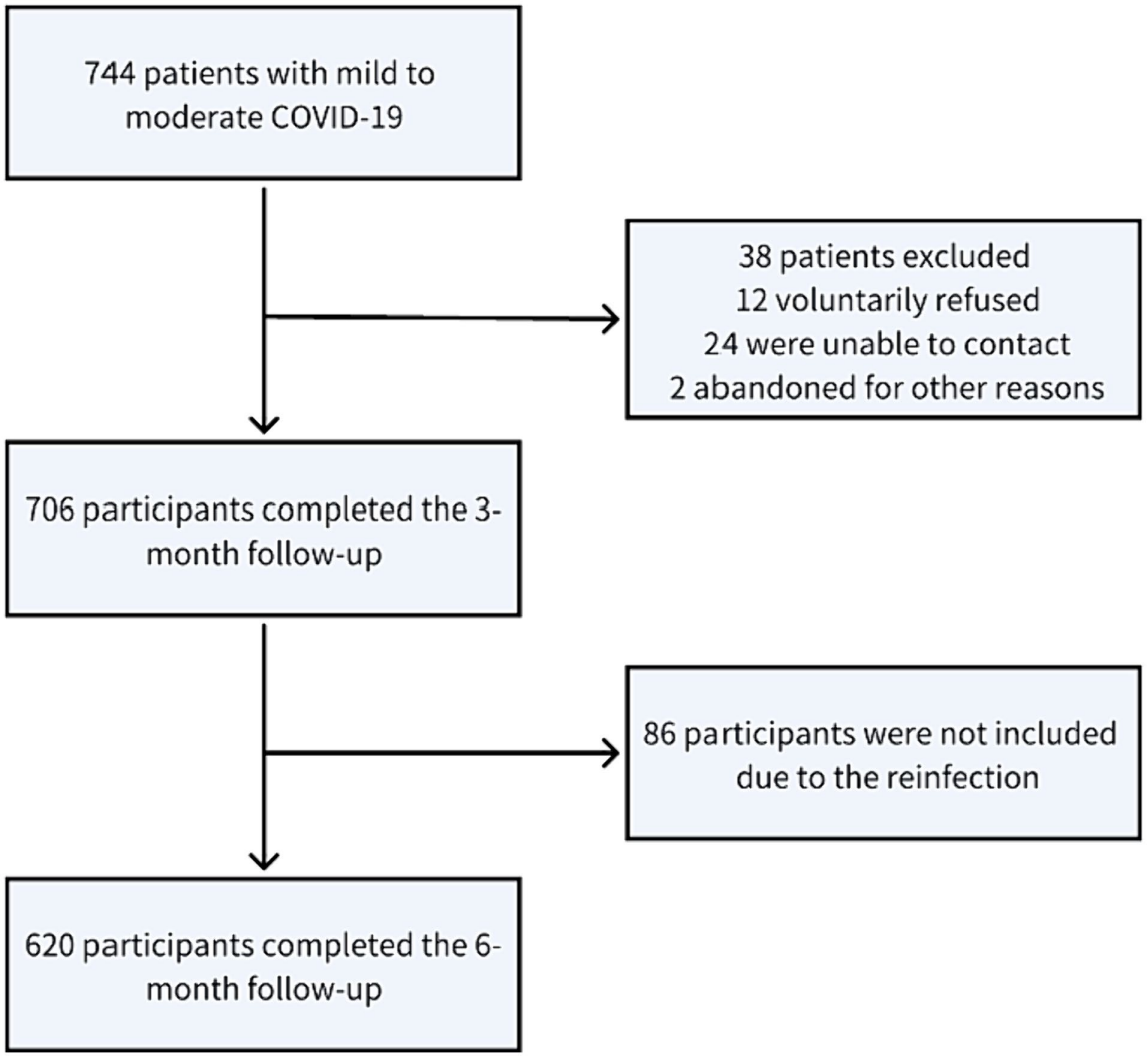
Flow chart of patients diagnosed with mild to moderate COVID-19 between December 1 and July 30, 2023.

In this study, a cohort of 744 patients, diagnosed with mild to moderate COVID-19, was observed from December 1, 2022, through July 30, 2023. However, the sample size was eventually reduced to 706 due to the exclusion of 38 patients. These exclusions were a result of a lack of follow-up participation: 12 patients voluntarily opted out, 24 were unreachable, and 2 withdrew due to miscellaneous reasons. After the 3 months follow-up period, all 706 participants remained in the study. However, at the 6 months mark, only 620 participants were included in the data analysis. The decrease in participant count was due to the exclusion of 86 individuals who experienced COVID-19 reinfection during the observation period. These demographic and participation details are illustrated in Figure 1.

Table 1 delineates the demographic and clinical attributes of participants, segregated based on the 3 months and 6 months follow-up data. The median participant age stands at 35 years (interquartile range: 28, 43 years), with an age range of 21 to 59 years. The gender distribution is predominantly female (85% in participants).

**Table 1:** Characteristics of enrolled patients.

Characteristic	Long COVID3				Long COVID6			
	Overall, *N* = 706^a^	NO, *N* = 223^a^	Yes, *N* = 483^a^	*p*-value^b^	Overall, *N* = 620^a^	NO, *N* = 303^a^	Yes, *N* = 317^a^	*p*-value^b^
Gender				0.010				0.026
Female	597 (85%)	177 (79%)	420 (87%)		522 (84%)	245 (81%)	277 (87%)	
Male	109 (15%)	46 (21%)	63 (13%)		98 (16%)	58 (19%)	40 (13%)	
Year	35 (28, 43)	33 (27, 41)	35 (29, 44)	0.010	35 (28, 44)	34 (28, 43)	36 (29, 45)	0.036
BMI	22.9 (20.8, 25.7)	22.7 (20.6, 25.8)	23.0 (20.9, 25.7)	0.388	23.0 (20.7, 25.8)	23.0 (20.7, 25.8)	22.9 (20.7, 25.8)	0.774
Culture				0.701				0.623
College lower	176 (25%)	52 (23%)	124 (26%)		160 (26%)	75 (25%)	85 (27%)	
College	413 (58%)	131 (59%)	282 (58%)		360 (58%)	175 (58%)	185 (58%)	
College higher	117 (17%)	40 (18%)	77 (16%)		100 (16%)	53 (17%)	47 (15%)	
Smoke_and_Drink				0.462				0.347
Both	25 (3.5%)	11 (4.9%)	14 (2.9%)		23 (3.7%)	12 (4.0%)	11 (3.5%)	
Drink	29 (4.1%)	11 (4.9%)	18 (3.7%)		24 (3.9%)	10 (3.3%)	14 (4.4%)	
None	634 (90%)	196 (88%)	438 (91%)		556 (90%)	276 (91%)	280 (88%)	
Smoke	18 (2.5%)	5 (2.2%)	13 (2.7%)		17 (2.7%)	5 (1.7%)	12 (3.8%)	
Basic_disease				0.027				0.001
No	464 (66%)	159 (71%)	305 (63%)		403 (65%)	212 (70%)	191 (60%)	
One	152 (22%)	44 (20%)	108 (22%)		136 (22%)	65 (21%)	71 (22%)	
Two	49 (6.9%)	15 (6.7%)	34 (7.0%)		44 (7.1%)	19 (6.3%)	25 (7.9%)	
Three or above	41 (5.8%)	5 (2.2%)	36 (7.5%)		37 (6.0%)	7 (2.3%)	30 (9.5%)	
Hypertension				0.178				0.538
NO	640 (91%)	207 (93%)	433 (90%)		558 (90%)	275 (91%)	283 (89%)	
Yes	66 (9.3%)	16 (7.2%)	50 (10%)		62 (10%)	28 (9.2%)	34 (11%)	
Diabetes				0.412				0.319
NO	674 (95%)	215 (96%)	459 (95%)		590 (95%)	291 (96%)	299 (94%)	
Yes	32 (4.5%)	8 (3.6%)	24 (5.0%)		30 (4.8%)	12 (4.0%)	18 (5.7%)	
Heart diseases				0.446				0.069
NO	698 (99%)	222 (100%)	476 (99%)		612 (99%)	302 (100%)	310 (98%)	
Yes	8 (1.1%)	1 (0.4%)	7 (1.4%)		8 (1.3%)	1 (0.3%)	7 (2.2%)	
Haematological conditions				>0.999				0.374
NO	701 (99%)	222 (100%)	479 (99%)		615 (99%)	302 (100%)	313 (99%)	
Yes	5 (0.7%)	1 (0.4%)	4 (0.8%)		5 (0.8%)	1 (0.3%)	4 (1.3%)	
COPD				>0.999				0.616
NO	703 (100%)	222 (100%)	481 (100%)		617 (100%)	301 (99%)	316 (100%)	
Yes	3 (0.4%)	1 (0.4%)	2 (0.4%)		3 (0.5%)	2 (0.7%)	1 (0.3%)	
Allergic rhinitis				0.714				0.575
NO	622 (88%)	195 (87%)	427 (88%)		550 (89%)	271 (89%)	279 (88%)	
Yes	84 (12%)	28 (13%)	56 (12%)		70 (11%)	32 (11%)	38 (12%)	
Asthma				0.387				0.024
NO	688 (97%)	219 (98%)	469 (97%)		605 (98%)	300 (99%)	305 (96%)	
Yes	18 (2.5%)	4 (1.8%)	14 (2.9%)		15 (2.4%)	3 (1.0%)	12 (3.8%)	
Gastrointestinal problems				0.075				0.015
NO	689 (98%)	221 (99%)	468 (97%)		604 (97%)	300 (99%)	304 (96%)	
Yes	17 (2.4%)	2 (0.9%)	15 (3.1%)		16 (2.6%)	3 (1.0%)	13 (4.1%)	
Oncological conditions				0.164				0.038
NO	691 (98%)	221 (99%)	470 (97%)		606 (98%)	300 (99%)	306 (97%)	
Yes	15 (2.1%)	2 (0.9%)	13 (2.7%)		14 (2.3%)	3 (1.0%)	11 (3.5%)	
Thyroid disease				0.090				0.099
NO	677 (96%)	218 (98%)	459 (95%)		593 (96%)	294 (97%)	299 (94%)	
Yes	29 (4.1%)	5 (2.2%)	24 (5.0%)		27 (4.4%)	9 (3.0%)	18 (5.7%)	
Kidney problems				>0.999				0.499
NO	704 (100%)	223 (100%)	481 (100%)		618 (100%)	303 (100%)	315 (99%)	
Yes	2 (0.3%)	0 (0%)	2 (0.4%)		2 (0.3%)	0 (0%)	2 (0.6%)	
Immune system diseases				>0.999				>0.999
NO	703 (100%)	222 (100%)	481 (100%)		617 (100%)	302 (100%)	315 (99%)	
Yes	3 (0.4%)	1 (0.4%)	2 (0.4%)		3 (0.5%)	1 (0.3%)	2 (0.6%)	
Osteoarthrosis				0.042				0.043
NO	642 (91%)	210 (94%)	432 (89%)		562 (91%)	282 (93%)	280 (88%)	
Yes	64 (9.1%)	13 (5.8%)	51 (11%)		58 (9.4%)	21 (6.9%)	37 (12%)	

a *n* (%); Median (IQR).

b Pearson’s Chi-squared test; Wilcoxon rank sum test; Fisher’s exact test.

In terms of educational attainment, the majority (58%) hold a university degree, trailed by individuals with education level below college (25%). The prevalence of underlying health conditions is relatively low, with only 5.8% of the participants having three or more such conditions. Allergic rhinitis tops the list of comorbidities (84 participants, 12%), succeeded by hypertension (66 participants, 9.3%), and osteoarthritis (64 participants, 9.1%).

Upon comparing COVID-19 patients with and without long-term sequelae during the 3 months follow-up, it was noted that the group with LC had a higher proportion of females (87% versus 79%; *p* = 0.010), older median age (35 years versus 33 years; *p* = 0.010), and greater comorbidity prevalence. However, there were no significant differences in BMI, educational background, and substance use habits such as smoking and drinking.

The 6 months follow-up for LC mirrored these findings. Notable differences in osteoarthritis, depression, and anxiety were observed between the 3 months and 6 months follow-ups. As the duration progressed, other conditions like asthma (*p* = 0.024), gastrointestinal issues (*p* = 0.015), and oncological conditions (*p* = 0.038) appeared to influence the long-term effects of COVID-19.

**Figure 2:**
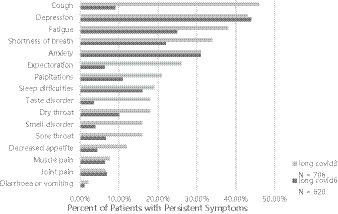
Outcomes of persistent symptoms 3- to 6 months after non-hospital patients admitted for acute COVID-19.

In the follow-up interview, 68.4% (483) of the participants reported experiencing at least one symptom after 3 months, while 49.7% (308) reported persistent symptoms after 6 months. The most prevalent symptoms at the 3 months mark were coughing (46%), fatigue (38%), and shortness of breath (34%). By the 6 months mark, the most frequent symptoms were fatigue (25%), shortness of breath (22%), and sleep disorders (16%). Anxiety and depression emerged as the most common symptoms during the follow-up period, particularly among participants with higher education levels and those with multiple comorbidities. Over time, most symptoms exhibited a declining trend, with the most notable reductions observed in coughing (from 46 to 9%), expectoration (from 26 to 6.3%), smell disorders (from 16 to 3.9%), and taste disorders (from 18 to 3.5%) (Figure 2).

**Figure 3:**
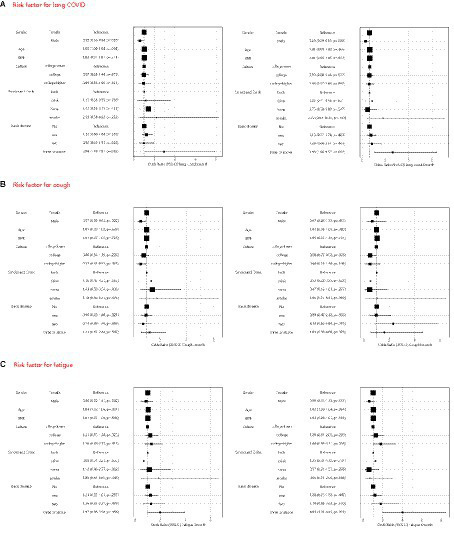

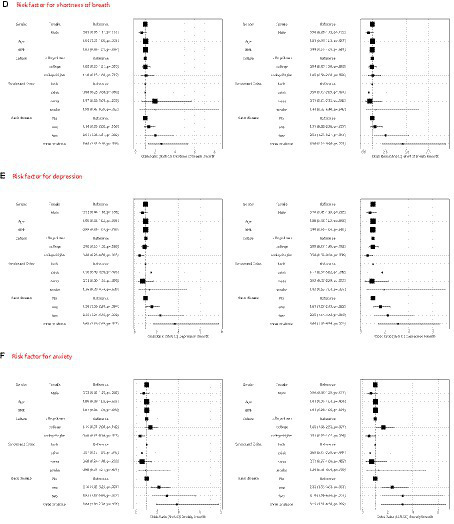
Multivariable logistic regression model to identify pre-existing risk factors for long COVID **(A)**, cough **(B)**, fatigue **(C)**, short of breath **(D)**, depression **(E)** and anxiety **(F)** in 3 months and 6 months.

In the multivariable regression analysis (Figure 3), the risk is lower for male and higher for female in 3 months (OR 0.58, 95% CI 0.36–0.94) and in 6 months (OR 0.49, 95% CI 0.29–0.83). In addition, participants with three or more comorbidities demonstrated an elevated risk in 3 months (OR 2.94, 95% CI 1.10–7.87) and in 6 months (OR 3.98, 95% CI 1.66–9.57). Factors such as age, BMI, level of education, and lifestyle habits like smoking and drinking did not significantly impact the risk of LC.

Common symptoms such as cough, fatigue, and shortness of breath were further examined using multivariate regression analysis. Males exhibit a reduced incidence of coughing over a 3 months observation period (OR 0.57, 95% CI 0.35–0.92), however, this difference effect dissipated after 6 months. Similarly, for males, experiencing fatigue or shortness of breath does not confer any advantages.

Compared to individuals with lower education levels, individuals with higher education levels are less likely to experience cough related symptoms within 3 months (OR 0.57, 95% CI 0.35–0.93). Correspondingly, compared to individuals with higher education levels and those with lower education levels, individuals with college levels have a lower risk of developing cough related symptoms within 6 months (OR 0.50, 95% CI 0.27–0.92). Age was identified as a risk factor for fatigue during the three-month follow-up (OR 1.04, 95% CI 1.02–1.06), while the presence of three or more comorbidities became more pronounced at the 6 months (OR 4.01, 95% CI 1.91–8.43).

With regard to shortness of breath, both two and three or more comorbidities were associated with an increased risk at both the 3 months and 6 months follow-ups.

Depression and anxiety are prevalent conditions among individuals with long COVID, and their prevalence does not significantly alter over time. During follow-up periods at 3 and 6 months, it has been observed that individuals with higher levels of education and those experiencing complications are at a greater risk of developing anxiety and depression. Conversely, factors such as age, gender, smoking habits, and alcohol consumption do not influence the occurrence of these mental health conditions.

## Discussion

In our research, we examined the enduring clinical consequences in non-hospitalized adult demographics following Omicron infection, while simultaneously scrutinizing alterations in symptom profiles and predisposing factors. Our findings revealed that a majority, exceeding half of the afflicted individuals, exhibited persistent symptoms. The most prevalent of these included cough, fatigue, dyspnea, and insomnia. Factors such as gender – specifically being female – and the presence of comorbidities (9–11) amplified the likelihood of developing post-COVID-19 conditions. Our analysis demonstrated that the risk of numerous health outcomes in the aftermath of mild to moderate COVID-19 infection became increasingly conspicuous within the initial 3 months post-infection, subsequently declining. The data also indicated that this risk fluctuated across different symptom spectra and evolved over time.

The prevailing symptoms observed amongst individuals include fatigue and shortness of breath, aligning with prior research (12–15). A meta-analysis has established that COVID-19 infection markedly elevates the likelihood of enduring fatigue and shortness of breath, with risk factors of 1.72 and 2.60 respectively, when assessed 4 weeks or more following initial infection, relative to a non-infected control cohort (16). This implies that regardless of the virus’s mutation or hospitalization status, chronic fatigue and shortness of breath persist as significant detriments impacting individuals’ quality of life. We further noted that while the severity of fatigue and shortness of breath diminished over time, the rate of decrease was gradual. Risk factors correlated with age and multiple comorbidities, but no significant relationship with gender was found. In our research, we did not explore the foundational mechanisms at play. However, contemporary scholarly work suggests that these mechanisms are likely complex and might encompass cerebral targeting, skeletal muscle impairment, as well as compromised erythrocyte functionality. Recent studies (17) reveal elevated levels of brain-reactive autoantibodies against MBP, MOG, tubulin, CP2, and synaptophysin in patients suffering from protracted COVID-19, suggesting a possible involvement of neuroautoimmune pathophysiology. Post-infection structural changes in skeletal muscle microvasculature due to immune response, such as reduced capillary density, thickened capillary basement membrane, and an increased number of CD169+ macrophages, may contribute to fatigue (18). Romy Kronstein-Wiedemann et al. (19) reported that long-term COVID-19 patients exhibited hindered oxygen-hemoglobin binding and enhanced carbon monoxide binding, indicating that persistent fatigue might be associated with compromised erythrocyte function in patients with prolonged coronavirus infection. Metabolic alterations in fatigued patients, including lactate, fumaric acid, symmetric dimethylarginine, and asymmetric dimethylarginine, could potentially serve as therapeutic targets (20, 21). Our findings also suggest a correlation between long-term COVID-19 and osteoarthrosis, implying possible involvement of the musculoskeletal system. This insight could aid in formulating rehabilitation strategies for managing post-COVID-19 fatigue (22).

During the course of a COVID-19 infection, numerous individuals reported symptoms such as coughing, expectoration, fatigue, shortness of breath, palpitations, and insomnia. However, it has been observed that the prevalence of these symptoms has exhibited a consistent decline over time. The most rapidly diminishing symptom appears to be coughing. This suggests that the manifestation of ‘Long-COVID’ symptoms tends to gradually diminish over time, which aligns with prior research on the progression of COVID-19 (23). In the current investigation, empirical data indicates that 30 days post onset or admission due to COVID-19, the estimated prevalence of cough was 18.6% (95% CI 10.6 to 30.7; 9 studies, *n* = 1,829). This figure saw a decrease to 8.6% (95% CI 5.3 to 13.7; 8 studies, *n* = 8,219) after a period of 90 days (24). A related study conducted by Osmanov et al. (25) revealed a decrease in fatigue levels in children from 15.8% at the time of discharge, to 8.8% seven months later. Furthermore, the percentage of sleep disturbances reported experienced a drop from 7.5 to 5.8%. Extensive research has suggested that gender significantly influences the clinical presentation and outcomes of various diseases, including those affecting respiratory functions. Our analysis of the available data delineates a robust correlation between gender and persistent post-COVID-19 cough, with females exhibiting a higher propensity towards experiencing this symptom as compared to males. Concurrently, our study also unearthed individuals boasting higher education levels demonstrated a reduced likelihood of suffering from a persistent cough, thereby suggesting that education might serve a protective role against this symptom (26). Intriguingly, our research did not unearth any significant correlation between smoking habits and the occurrence of a persistent cough. Despite the fact that smoking can unquestionably exacerbate respiratory health, it seemingly bears minimal influence on the persistence or severity of a cough specifically associated with COVID-19. This insinuates the possibility of unique mechanisms triggering coughs caused by this virus. The heightened activity of transient receptor potential (TRP) channels, which are expressed on the C fibers of the vagal nerve and mediate cough responses, as well as laryngeal hypersensitivity and dysfunction accompanied by abnormal vocal cord movement could potentially explain this phenomenon (27). Additionally, mast cells, known for expressing female sex hormone receptors, may shed light on the cause of persistent cough in females.

An exhaustive examination of accessible data unveils a significant prevalence of anxiety and depression amongst individuals suffering from long COVID, corroborating prior research (28–31). These psychiatric manifestations may endure well beyond the resolution of the disease’s acute phase, resulting in considerable distress and compromised functionality (32). Elements such as ambiguity associated with long COVID, physical manifestations, and social segregation contribute to the inception and intensification of anxiety and depression within this demographic. Intriguingly, our investigation underscores a significant correlation between elevated education levels and heightened incidence of anxiety and depression amongst long COVID patients. Despite education typically providing individuals with superior tools to tackle health-related adversities, it may also precipitate excessive introspection, escalated health apprehension, and an illusion of control over health consequences. The impetus to excel acadically can amplify pre-existing psychological susceptibilities. These observations emphasize the need for custom-made interventions targeting individuals with advanced education to cater to their distinct mental health requirements. Our analysis further delves into the repercussions of multiple medical complications on anxiety and depression amongst long COVID patients. Individuals with co-morbidities or a history of numerous complications are at an escalated risk for the development of psychological distress. The obligation of managing intricate medical conditions, confronting ambiguity regarding recuperation, and grappling with an extended illness trajectory can contribute to exacerbated anxiety and depression. This insight underscores the indispensability of comprehensive care that addresses both physical and mental health dimensions for long COVID patients with multiple complications.

### Limitations

This cohort study possesses several inherent limitations. Primarily, it is important to note the disproportionate representation of women in the COVID-19 lifeline cohort which may introduce selection bias, compared to the broader lifeline population. Secondly, the absence of a control group, composed of healthy adults unaffected by COVID-19, restricts the comparative scope of our study the third limitation arises from potential comorbidities or complications that some patients might encounter during the follow-up period. These additional health issues could potentially influence both their overall health status and the persistence and prevalence of COVID-19 symptoms. The fourth limitation pertains to the subjective nature of patient-reported outcomes, such as fatigue, anosmia, and dysgeusia. These self-reported symptoms may not be as precise or consistent as a physician’s clinical diagnosis. Lastly, we cannot disregard the possibility of behavioral and environmental disparities between infected and uninfected individuals. Such differences could potentially inflate the calculated incidence rate among those infected with COVID-19.

## Conclusion

Following the COVID-19 pandemic, it was observed that the majority of patients’ symptoms gradually subsided, with cough, expectoration, olfactory disturbance, and gustatory disorder showing the most rapid decline. However, after a 6 months observation period, nearly half of the affected individuals continued to exhibit at least one symptom. Predominantly, fatigue, dyspnea, and sleep disturbances were the most frequently reported post-illness conditions. The risk factors associated with these residual symptoms varied slightly. For instance, cough was predominantly observed in women, establishing gender as a principal risk factor for this symptom. Age and pre-existing health conditions were more frequently linked to fatigue and shortness of breath. Furthermore, psychological disorders such as depression and anxiety were prevalent among the post-COVID-19 conditions. Currently, the mechanisms underlying these diverse post-COVID-19 symptoms remain elusive. Future research should aim to devise treatment strategies tailored to these specific symptoms to enhance therapeutic efficacy.

## Data availability statement

The original contributions presented in the study are included in the article/Supplementary material, further inquiries can be directed to the corresponding author.

## Ethics statement

The studies involving humans were approved by the Ethics Committee of Beijing Longfu Hospital. The studies were conducted in accordance with the local legislation and institutional requirements. The participants provided their written informed consent to participate in this study.

## Author contributions

WR: Writing – original draft. JQ: Writing – review & editing. WT: Writing – review & editing. YY: Data curation, Writing – original draft. JY: Data curation, Writing – original draft. PK: Methodology, Writing – original draft.

## Footnotes

^1^ https://cran.r-project.org
