# Supplementary material for: Long COVID outcomes following omicron wave in non-hospital population
Source: Front Public Health. 2024 Mar 15;12:1377866. doi: 10.3389/fpubh.2024.1377866 (PMC10978792; doi:10.3389/fpubh.2024.1377866)
Supplement: Supplementary file 1 [file Data_Sheet_1.PDF]

PARTICIPANT ID I__I I__I I__I I__I I__I -- I___I I___I I___I I___I

Module 1: Background demographical and epidemiological information

Date of module 1 completion: [_D_][_D_]/[_M_][_M_]/[_Y_][_Y_][_Y_][_Y_]

1.1 Demographics

Sex at Birth: Male Female **Age:** ____years;
Height ___ cm **Weight:** ___kg
BMI:_____
level of education college lower college college higher
Smoking and drinking: smoking drinking both none

1.2 Pre-existing conditions in the year prior to your acute illness of COVID-19:

In the year prior to the acute illness of COVID-19, has the participant been diagnosed with any of the following conditions?

**Hypertension:**	Yes No Unknown;
**Diabetes:**	Yes No Unknown;
**Heart diseases:**	Yes No Unknown;
**Haematological conditions**:	Yes No Unknown;
**COPD:**	Yes No Unknown;
**Allergic rhinitis:**	Yes No Unknown;
**Asthma:**	Yes No Unknown;
**Gastrointestinal problems:**	Yes No Unknown;
**Oncological conditions:**	Yes No Unknown;
**Thyroid disease:**	Yes No Unknown;
**Kidney problems:**	Yes No Unknown;
**Immune system diseases:**	Yes No Unknown.
**Osteoarthrosis:**	Yes No Unknown;

1.3Diagnosis of acute illness of COVID-19 (first episode, in case of re-infection)

Date of onset of symptoms of **acute** COVID-19: [_D_][_D_]/[_M_][_M_]/[_Y_][_Y_][_Y_][_Y_];
Did the participant receive a **diagnosis** of COVID-19 by a health care worker during the **acute illness**?
Yes No Unknown;
Did the participant have a **diagnostic test?** Yes No
Unknown; If yes, complete the 3 questions below:
Did the participant have a **PCR test** during the acute illness?
Yes, positive Yes, negative Not performed Unknown;
If positive, date of positive PCR test: [_D_][_D_]/[_M_][_M_]/[_Y_][_Y_][_Y_][_Y_]
Did the participant have an **antigen test** (rapid test) during acute illness?
Yes, positive Yes, negative Not performed Unknown;
If positive, date of positive antigen test:
[_D_][_D_]/[_M_][_M_]/[_Y_][_Y_][_Y_][_Y_]

PARTICIPANT ID I____I I___I I___I I___I I___I – I___I I___I I___I I___I

Module 2. Follow up interview

Date of follow up interview: [_D_][_D_]/[_M_][_M_]/[_Y_][_Y_][_Y_][_Y_]
Re-infection Yes No

2.1 Incidence of symptoms after acute illness of COVID-19 in 3 month

Did the participant experience any of the following symptoms after the acute illness of COVID-19 that were **not experienced** before the acute episode of COVID-19? Yes No Unknown; **If yes**, please respond to questions below:

**Cough:**	Yes No Unknown;
**Expectoration:**	Yes No Unknown;
**Sore throat:**	Yes No Unknown;
**Dry throat**:	Yes No Unknown;
**Fatigue:**	Yes No Unknown;
**Sleep difficulties:**	Yes No Unknown;
**Decreased appetite:**	Yes No Unknown;
**Smell disorder:**	Yes No Unknown;
**Taste disorder:**	Yes No Unknown;
**Palpitations:**	Yes No Unknown;
**Shortness of breath:**	Yes No Unknown;
**Joint pain:**	Yes No Unknown.
**Muscle pain:**	Yes No Unknown;
**Diarrhoea or vomiting:**	Yes No Unknown;

2.2 The Self-Rating Depression Scale(SDS) Score:_____

1. I feel depressed and depressed... ① Rarely ② Sometimes ③ Often ④ Continuously
2. I think mornings are the best of the day... ① Rarely ② Sometimes ③ Often ④ Continuously
3. Always crying inexplicably or feeling like crying... ① Rarely ② Sometimes ③ Often ④ Continuously
4. I don't sleep well at night... ① Rarely ② Sometimes ③ Often ④ Continuously
5. I eat as much as usual... ① Rarely ② Sometimes ③ Often ④ Continuously
6. I feel as happy as ever when I have close contact with the opposite sex. ① Rarely ② Sometimes ③ Often ④ Continuously
7. I feel like my weight is losing... ① very little ② sometimes ③ often ④ continuously
8. I have the annoyance of constipation... ① rarely ② sometimes ③ often ④ continuously
9. I feel like my heart is beating faster than usual... ① Rarely ② Sometimes ③ Often ④ Continuously
10. I feel fatigued for no reason... ① Rarely ② Sometimes ③ Often ④ Continuously
11. My mind is as clear as usual... ① Rarely ② Sometimes ③ Often ④ Continuously
12. I don't feel any difficulty doing things as usual... ① Rarely ② Sometimes ③ Often ④ Continuously
13. I feel restless and find it difficult to stay calm... ① Rarely ② Sometimes ③ Often ④ Continuously
14. I feel hopeful for the future... ① Rarely ② Sometimes ③ Often ④ Continuously
15. I am more easily angry and excited than usual... ① Rarely ② Sometimes ③ Often ④ Continuously
16. I think making a decision is an easy task... ① Rarely ② Sometimes ③ Often ④ Continuously
17. I think I am a useful person, and others need me very little, sometimes, often, and continuously
18. My life is very meaningful... ① rarely ② sometimes ③ often ④ continuously
19. I think if I die, others will live better... ① Rarely ② Sometimes ③ Often ④ Continuously
20. I am still interested in things that I am usually interested in... ① Rarely ② Sometimes ③ Often ④ Continuously

2.3 The Self-Rating Anxiety Scale (SAS) Score:_____

1. I feel more nervous and anxious than usual ① rarely ② sometimes ③ often ④ persistently
2. Feeling scared for no reason ① rarely ② sometimes ③ often ④ continuously
3. Easy to feel agitated or frightened ① rarely ② sometimes ③ often ④ persistently
4. I feel like I might go crazy ① rarely ② sometimes ③ often ④ persistently
5. I feel that everything is good and nothing unfortunate will happen ① rarely ② sometimes ③ often ④ continuously
6. Hands and feet tremble and tremble ① rarely ② sometimes ③ frequently ④ continuously
7. Suffering from headaches, head and neck pain, and back pain ① Rarely ② Sometimes ③ Often ④ Continuously
8. Feeling easily weakened and fatigued ① rarely ② sometimes ③ often ④ persistently
9. Feeling calm and easy to sit quietly ① rarely ② sometimes ③ often ④ continuously
10. I feel that my heart beats very fast ① rarely ② sometimes ③ often ④ continuously
11. Suffering from waves of dizziness ① Rarely ② Sometimes ③ Often ④ Continuously
12. Has fainting attacks or feels like fainting ① Rarely ② Sometimes ③ Often ④ Continuously
13. It feels easy to inhale and exhale ① rarely ② sometimes ③ frequently ④ continuously
14. Numbness and stinging in hands and feet ① rarely ② sometimes ③ frequently ④ persistently
15. Suffering from stomach pain and indigestion ① Rarely ② Sometimes ③ Often ④ Continuously
16. Frequent need to urinate ① rarely ② sometimes ③ frequently ④ continuously
17. Hands are often dry and warm ① rarely ② sometimes ③ often ④ continuously
18. Blushing and fever ① rarely ② sometimes ③ frequently ④ persistently
19. Easy to fall asleep and sleep well ① rarely ② sometimes ③ often ④ continuously
20. Nightmares ① rare ② sometimes ③ frequent ④ persistent

PARTICIPANT ID I____I I___I I___I I___I I___I – I___I I___I I___I I___I

Module 2. Follow up interview

Date of follow up interview: [_D_][_D_]/[_M_][_M_]/[_Y_][_Y_][_Y_][_Y_]
Re-infection Yes No

3.1 Incidence of symptoms after acute illness of COVID-19 in 6 month

Did the participant experience any of the following symptoms after the acute illness of COVID-19 that were **not experienced** before the acute episode of COVID-19? Yes No Unknown; **If yes**, please respond to questions below:

**Cough:**	Yes No Unknown;
**Expectoration:**	Yes No Unknown;
**Sore throat:**	Yes No Unknown;
**Dry throat**:	Yes No Unknown;
**Fatigue:**	Yes No Unknown;
**Sleep difficulties:**	Yes No Unknown;
**Decreased appetite:**	Yes No Unknown;
**Smell disorder:**	Yes No Unknown;
**Taste disorder:**	Yes No Unknown;
**Palpitations:**	Yes No Unknown;
**Shortness of breath:**	Yes No Unknown;
**Joint pain:**	Yes No Unknown.
**Muscle pain:**	Yes No Unknown;
**Diarrhoea or vomiting:**	Yes No Unknown;

3.2 The Self-Rating Depression Scale(SDS) Score:_____

1. I feel depressed and depressed... ① Rarely ② Sometimes ③ Often ④ Continuously
2. I think mornings are the best of the day... ① Rarely ② Sometimes ③ Often ④ Continuously
3. Always crying inexplicably or feeling like crying... ① Rarely ② Sometimes ③ Often ④ Continuously
4. I don't sleep well at night... ① Rarely ② Sometimes ③ Often ④ Continuously
5. I eat as much as usual... ① Rarely ② Sometimes ③ Often ④ Continuously
6. I feel as happy as ever when I have close contact with the opposite sex. ① Rarely ② Sometimes ③ Often ④ Continuously
7. I feel like my weight is losing... ① very little ② sometimes ③ often ④ continuously
8. I have the annoyance of constipation... ① rarely ② sometimes ③ often ④ continuously
9. I feel like my heart is beating faster than usual... ① Rarely ② Sometimes ③ Often ④ Continuously
10. I feel fatigued for no reason... ① Rarely ② Sometimes ③ Often ④ Continuously
11. My mind is as clear as usual... ① Rarely ② Sometimes ③ Often ④ Continuously
12. I don't feel any difficulty doing things as usual... ① Rarely ② Sometimes ③ Often ④ Continuously
13. I feel restless and find it difficult to stay calm... ① Rarely ② Sometimes ③ Often ④ Continuously
14. I feel hopeful for the future... ① Rarely ② Sometimes ③ Often ④ Continuously
15. I am more easily angry and excited than usual... ① Rarely ② Sometimes ③ Often ④ Continuously
16. I think making a decision is an easy task... ① Rarely ② Sometimes ③ Often ④ Continuously
17. I think I am a useful person, and others need me very little, sometimes, often, and continuously
18. My life is very meaningful... ① rarely ② sometimes ③ often ④ continuously
19. I think if I die, others will live better... ① Rarely ② Sometimes ③ Often ④ Continuously
20. I am still interested in things that I am usually interested in... ① Rarely ② Sometimes ③ Often ④ Continuously

2.3 The Self-Rating Anxiety Scale (SAS) Score:_____

1. I feel more nervous and anxious than usual ① rarely ② sometimes ③ often ④ persistently
2. Feeling scared for no reason ① rarely ② sometimes ③ often ④ continuously
3. Easy to feel agitated or frightened ① rarely ② sometimes ③ often ④ persistently
4. I feel like I might go crazy ① rarely ② sometimes ③ often ④ persistently
5. I feel that everything is good and nothing unfortunate will happen ① rarely ② sometimes ③ often ④ continuously
6. Hands and feet tremble and tremble ① rarely ② sometimes ③ frequently ④ continuously
7. Suffering from headaches, head and neck pain, and back pain ① Rarely ② Sometimes ③ Often ④ Continuously
8. Feeling easily weakened and fatigued ① rarely ② sometimes ③ often ④ persistently
9. Feeling calm and easy to sit quietly ① rarely ② sometimes ③ often ④ continuously
10. I feel that my heart beats very fast ① rarely ② sometimes ③ often ④ continuously
11. Suffering from waves of dizziness ① Rarely ② Sometimes ③ Often ④ Continuously
12. Has fainting attacks or feels like fainting ① Rarely ② Sometimes ③ Often ④ Continuously
13. It feels easy to inhale and exhale ① rarely ② sometimes ③ frequently ④ continuously
14. Numbness and stinging in hands and feet ① rarely ② sometimes ③ frequently ④ persistently
15. Suffering from stomach pain and indigestion ① Rarely ② Sometimes ③ Often ④ Continuously
16. Frequent need to urinate ① rarely ② sometimes ③ frequently ④ continuously
17. Hands are often dry and warm ① rarely ② sometimes ③ often ④ continuously
18. Blushing and fever ① rarely ② sometimes ③ frequently ④ persistently
19. Easy to fall asleep and sleep well ① rarely ② sometimes ③ often ④ continuously
20. Nightmares ① rare ② sometimes ③ frequent ④ persistent
